# Serum Dioxin-like Activity Is Associated with Reproductive Parameters in Young Men from the General Flemish Population

**DOI:** 10.1289/ehp.9224

**Published:** 2006-07-27

**Authors:** Willem Dhooge, Nicolas van Larebeke, Gudrun Koppen, Vera Nelen, Greet Schoeters, Robert Vlietinck, Jean-Marc Kaufman, Frank Comhaire, for the Flemish Environment and Health Study Group

**Affiliations:** 1 Department of Endocrinology, University Hospital Ghent, Belgium; 2 Study Centre for Carcinogenesis and Primary Prevention of Cancer, Department of Radiotherapy, Nuclear Medicine and Experimental Cancerology, University Hospital Ghent, Belgium; 3 Flemish Institute of Technological Research (VITO), Centre of Expertise in Environmental Toxicology, Mol, Belgium; 4 Provincial Institute for Hygiene, Antwerp, Belgium; 5 Department of Human Heredity, Catholic University of Leuven, Leuven, Belgium

**Keywords:** dioxin, DR-CALUX, egg consumption, hormones, male fertility, semen volume, sperm concentration, TCDD, testosterone

## Abstract

**Background:**

2,3,7,8-Tetrachlorodibenzo-*p*-dioxin (TCDD) and some related environmental contaminants are aryl hydrocarbon receptor (AhR) ligands that exert reproductive and developmental toxicity in laboratory animals. In humans, fertility-related effects are less documented.

**Objective:**

The aim of this study was to investigate the relationship between dioxin-like biological activity in serum and parameters of reproductive status in men from the general population 5 months after a polychlorinated biphenyl and dioxin food-contamination episode in Belgium.

**Design:**

In the framework of the cross-sectional Flemish Environment and Health Study (FLEHS), we recruited 101 men 20–40 years of age and evaluated sperm parameters, measured sex hormones, and gathered information on a number of lifestyle factors. In addition, we determined the AhR-mediated enzymatic response elicited by individual serum samples and expressed it as TCDD equivalent concentrations (CALUX-TEQs) using an established transactivation assay.

**Results:**

Age (*p* = 0.04) and the frequency of fish (*p* = 0.02) and egg (*p* = 0.001) consumption were independent positive determinants of serum dioxin-like activity. After correcting for possible confounders, we found that a 2-fold increase in CALUX-TEQ > 16 pg/L was associated with a 7.1% and 6.8% (both *p* = 0.04) decrease in total and free testosterone, respectively. We also observed a more pronounced drop in semen volume of 16.0% (*p* = 0.03), whereas sperm concentration rose by 25.2% (*p* = 0.07). No relationship was found with total sperm count or sperm morphology.

**Conclusions:**

These data suggest an interaction of dioxin-like compounds with the secretory function of the seminal vesicles or prostate, possibly indirectly through an effect on testosterone secretion, at levels not affecting spermatogenesis as such.

2,3,7,8-Tetrachlorodibenzo-*p*-dioxin (TCDD) is generally considered to be the most potent man-made biological agent. In laboratory animals, changes in multiple endocrine and growth factor systems have been reported in a manner that is tissue, sex, and age dependent (Birnbaum and Tuomisto 2000). In humans, environmental exposure has been linked with the development of insulin resistance and diabetes (Kern et al. 2004), a decreased male to female sex ratio of the offspring (Mocarelli et al. 2000), and alterations of reproductive hormones in men (Egeland et al. 1994).

Most, if not all, of the described negative health effects of TCDD are believed to be mediated by the sustained activation of the aryl hydrocarbon receptor (AhR) expressed in many tissues in the body (Safe 1995). A number of other compounds, including certain polychlorinated dibenzodioxins (PCDDs), polychlorinated dibenzofurans (PCDFs), and polychlorinated biphenyls (PCBs) exhibit similar AhR-mediated biological responses. Traditionally, the potency of these substances relative to TCDD has been expressed as a consensus toxic equivalency factor (TEF), allowing the translation of analytically determined single concentrations of chemicals in a sample into a biological activity measure—the TCDD equivalent concentration (TEQ)—assuming a model of dose additivity (Safe 1990; van den Berg et al. 2000). In laboratory experiments, Hamm et al. (2003) observed reproductive effects similar to those described for TCDD for a number of AhR ligands or mixtures.

PCDDs and PCDFs are widespread environmental contaminants, formed as byproducts in certain industrial production processes or during combustion at insufficiently controlled temperatures (Safe 1990). PCBs, commercial products whose manufacture and application was stopped following emerging scientific evidence of their toxicologic effects in humans and laboratory animals [Agency for Toxic Substances and Disease Registry (ATSDR) 2000], still enter the environment through accidental spillage. Humans and wildlife are exposed to these polychlorinated compounds, basically through the food chain. People’s internal levels of these compounds vary with the source of contamination, the individual’s food habits, and his/her polymorphic nature in toxicokinetics (ATSDR 2000; Aylward et al. 2005; Safe 1995). The use of biomarkers of exposure, such as serum levels of dioxins, can eliminate part of the problem posed by the inter-individual internal exposure heterogeneity that exists in exposed or control populations (Staessen et al. 2001).

Only a limited number of PCBs are AhR ligands, whereas the biological effects of other congeners have been linked to their estrogenic, antiandrogenic, or thyroid-disruptive potential (ATSDR 2000). The resulting complexity of the organochlorine mixtures present in the environment, both in view of compound number as well as of the multitude and sometimes interactive nature of the affected physiologic processes in the body, complicate the elucidation of exposure–effect links, leading to contradicting results in different epidemiologic studies (Toft et al. 2004). As an alternative to the traditional chemical analytical assessment, cellular assays have been developed that measure dioxin-like activity, integrating the biological response of all compounds capable of binding to the AhR in environmental samples and controlling for possible nonadditive effects (Focant et al. 2005; Koppen et al. 2001).

Reproductive and endocrine effects of neonatal and gestational exposure are among the most sensitive end points of dioxin toxicity (Kogevinas 2001). However, effects of exposure at older age are less pronounced, and consequently epidemiologic evidence of a relationship between dioxin-like substances and impaired endocrine or fertility status in humans is limited. In 1999, a project consortium performed a feasibility study of biomarkers in humans of three age categories living in an urban or a rural region in Flanders (FLEHS project) (Staessen et al. 2001). In the adolescent cohort, a significant relationship between chemical-activated luciferase expression–TCDD equivalent concentrations (CALUX-TEQ) and delayed puberty in girls was found, as estimated by Tanner breast stage, but not pubic hair stage (Den Hond et al. 2002; Staessen et al. 2001). In view of the alleged negative time trends in sperm quality, possibly mediated by environmental factors (Swan et al. 2000), we wished to investigate the role of dioxins in male reproductive health several months after a 1999 incident of contamination of the food chain with PCBs and dioxins in Belgium (Bernard et al. 1999). We restricted the age range of participants to 20–40 years to exclude, as much as possible, age-related endocrine alterations in the hypothalamo–pituitary–gonadal axis (Kaufman and Vermeulen 2005).

## Methods

### Subjects

Short questionnaires on diet, lifestyle, and profession with an accompanying letter to 2,487 possible male candidates, selected randomly from the municipal population registries of Antwerp and Peer, Belgium. After ordering all male inhabitants as a function of date of birth, every *x*th man was included to obtain a first selection of the potential participants with ages evenly distributed between 20 and 40 years. The overall response rate was 30%. Of the 744 responders, 207 men were eligible after consideration of the following exclusion criteria: vasectomy, professional exposure to possible threats for male reproductive health (chemical industry, dry cleaning, production, military airport), working in a different environmental setting than the home address (rural vs. urban), and commuting long distances. Eligible candidates were contacted by telephone to check their willingness to participate and provide further details regarding semen sample collection and minimal hygienic conditions. They were asked to carefully report the hour of semen collection and the abstinence period; interviewers stressed that subjects should abstain from ejaculation for preferably 3 days. Overall, 55% of the eligible men agreed to participate, and 101 men were allowed to enter the study, with preference given to nonsmokers. No differences in age or residence period were noted between those who entered the study and those who did not. Of the participants with proven fertility, 56% were fathers of an average of two children; this was not different from the rest of the responders. All participants came to the study site and gave their informed consent. The ethics committee of the Catholic University of Leuven approved the study.

### Procedures and methods

Semen samples were collected by masturbation and processed within 45 min at the study site. Trained nurses prepared a seminal smear and added 2 mL Hayem’s anticoagulant and preservative solution, and the specimen was stored at 4°C. All samples were sent to the andrology department within 4 days of semen collection. Ejaculate volumes were estimated using a graduated pipette. A single technician scored sperm concentration in duplicate using disposable counting chambers (Cellvision, Heerhugowaard, the Netherlands), and determined sperm morphology on air-dried Papanicolaou-stained seminal smears. Using the morphology method recommended by the 1992 World Health Organization manual (WHO 1992), only sperm with absolutely no defects were classified as normal. The total sperm count (TSC) was derived by multiplying the individual’s sperm concentration and volume. Each participant completed a questionnaire designed to assess lifestyle, social class, use of tobacco and alcohol, food intake, and intake of medicines, similar to the questionnaire used in the adolescent study (Staessen et al. 2001). Nonsmoking participants were classified as ex-smokers if they had smoked at least one cigarette per day for at least 1 year; otherwise they were classified as never-smokers. The amount of animal fat intake per person was calculated from their intake of meat, fish, and dairy products in the year before study, by use of Dutch food composition tables (Stichting Nevo 1993).

All tests were performed in specialized laboratories that met national and international quality control standards. Venous blood was obtained and centrifuged at the site. Serum was aliquoted for determination of markers of hormone status and dioxin exposure in plastic or glass tubes, respectively, and stored at −20°C until analysis. We used commercial immuno-assays to determine serum levels of total testosterone (Medgenix, Fleurus, Belgium), luteinizing hormone (LH), follicle-stimulating hormone (FSH) (Roche Diagnostics, Vilvoorde, Belgium), sex hormone-binding globulin (SHBG; Orion Diagnostica, Espoo, Finland), total 17β-estradiol (E_2_; Clinical Assay; DiaSorin srl, Saluggia, Italy), and inhibin B (Serotec, Oxford, UK). The free fraction of testosterone (fT) was calculated from serum testosterone and SHBG, assuming a fixed albumin concentration (Vermeulen et al. 1999). The intra- and interassay coefficients of variation for all assays were < 12%. For every individual, we calculated the testosterone/LH ratio.

Assessment of dioxins by direct chemical measurement requires ≤ 50 mL blood. Therefore, we measured dioxin-like activity in serum using the dioxin receptor (DR, or AhR)-driven Chemical-Activated LUciferase eXpression (DR-CALUX) in cultured H4IIE cells, as described in detail elsewhere (Koppen et al. 2001; Murk et al. 1996). Briefly, after *n*-hexane extraction of 2 mL blood serum and removal of matrix components by passage through a 33% H_2_SO_4_ silica column, extracts were quantitatively transferred to a conical vial for evaporation until almost dry. Dimethyl sulfoxide (DMSO; 7.5 μL; Acros Organics, Geel, Belgium) was added, and the extract was diluted to a total volume of 750 μL with minimal essential medium (Gibco, Merelbeke, Belgium).

Rat hepatoma H4IIE cells, stably transfected with an AhR-controlled luciferase reporter gene construct, were grown in 96-well plates in minimal essential medium with 10% fetal calf serum (Gibco) at 37°C and 5% CO_2_. At 70–80% confluency, cells were treated with 100 μL of the sample extract in triplicate and then incubated at 37°C. After 24 hr, cells were washed, lysed, and stored at −80°C for at least 1 hr.

Luciferase activity was measured on a Victor 2 Luminometer (EG&G Wallac, Oosterhout, the Netherlands). Samples were read on a standard curve of TCDD (0.3–100 pmol/L) that was included in each 96-well plate. The method thus yields a biological measure of the combined presence of chemicals with an activity equivalent to an amount of the reference compound TCDD. Results were expressed in nanograms of CALUX-TEQs per liter serum or per gram serum fat, determined enzymatically. The limit of detection was calculated as the signal measured from the DMSO solvent control on each well plate plus three times its SD. However, this limit of detection varied with cell growth and volume of the blood samples (Koppen et al. 2001) and ranged between 2 and 10 ng/L during the FLEHS experiments. A fetal calf serum sample was run for each series of study samples as internal standard. The interexperiment variation was < 30% and accepted as normal for this low-loaded sample.

After logarithmic transformation, CALUX-TEQs expressed per volume serum exhibited a bimodal distribution with a clear intermode nadir at 16 pg/L (coinciding with the 10th percentile). This artifact was probably related to the uncertainty of the DR-CALUX assay results at or near the limit of detection than to the existence of a threshold value of dioxin activity.

### Statistical analyses and exclusions

Hormone levels are reported as molar units or international units per liter of serum except for inhibin B which is expressed in nanograms per liter. One participant had a hormone and sperm profile suggestive of Klinefelter syndrome and was excluded from the dataset, thus reducing the number of participants to 100. Normal data are described as arithmetic mean ± SD. Data that were not normally distributed are described by median and interquartile range (IQR) or log-transformed and described by geometric mean (GM) and 95% confidence intervals (CIs) in case of correction for confounding variables. We investigated the relationships between CALUX-TEQs and biomarkers of effect using scatter plots and Spearman’s rank correlation (*r*), and subsequently calculated dose–effect relations in individuals by use of multiple-linear regression. We calculated odds ratios (ORs) for a disorder using multiple logistic regression. Subjects were studied in two separate periods: late June and early September. This confounded the relationships between CALUX-TEQs and biomarkers of effect and was accounted for in the regression analyses. Hormones were corrected for relevant confounders, irrespective of statistical significance. Because of the nonlinear relationship between sperm parameters and abstinence period, the latter was coded < 2 days, between 2 and 4 days, or between 4 and 6 days or higher, and corrected for in multiple linear regression. Age was included as a confounder in sperm parameter analyses (Kidd et al. 2001). Correlation and regression analyses of CALUX-TEQ expressed per volume serum are reported here, but similar results were obtained when CALUX-TEQs were expressed per gram of serum fat. Although cubic or square root conversion was necessary to obtain normally distributed residuals for a number of dependent variables, we used the logarithmic transformation. Although this lowered the statistical significance of the model, it has the advantage of relevance and interpretability of the resulting regression coefficients or adjusted means while residues remained within acceptable boundaries of compliance with statistical theory. Effect sizes were calculated from linear regression coefficients for a 2-fold increase in the biomarker of dioxin exposure. Statistical analyses were performed with SPSS software (version 12.0; SPSS Inc., Chicago, IL, USA).

## Results

### Participant’s characteristics and reproductive parameters

The characteristics of the population are presented in Table 1. Blood was collected between 0930 and 2000 hours (mean ± SD = 15.07 ± 03.28 hr). Before and after correction for age and time of day blood was sampled, the eight obese participants [body mass index (BMI) > 30 kg/m^2^] had lower testosterone levels [GM (95% CI), 10.2 nmol/L (8.3–12.4) versus 14.4 nmol/L (13.6–15.3); *p* = 0.001] and higher E_2_ levels [95.0 pmol/L (78.3–115.2) versus 65.7 pmol/L (62.1–69.5); *p* < 0.001] than the other participants, but no differences between these two groups were seen for any sperm parameter or dioxin value. Education level or reported intake of medication was not associated with any hormone or sperm parameter; however, participants with a history of serious illness or surgery had a drastically lower total sperm count before and after correction for age, completeness of the semen sample, and abstinence period [GM (95% CI), 38.4 × 10^6^/mL (20.3–73.0) vs. 117.7 × 10^6^/mL (95.1–145.8); *p* = 0.02].

The 31 ex-smokers had stopped smoking for a median (IQR) of 6.2 years (2.3–12.4). Former duration of smoking and self-reported hours per day of passive smoking did not correlate with any sperm or hormone parameter. The sperm and hormone values of the two current smokers were well within the normal range of the total population. The calculated amount of alcohol consumed (grams per day) was not related to any hormone or sperm parameter. Semen volume correlated significantly negatively with sperm concentration (*r* = −0.35, *p* < 0.001). Semen volume and total sperm count, but not sperm concentration, correlated positively with duration of abstinence (*r* for semen volume = 0.35, *p* < 0.001; TSC = 0.25, *p* = 0.01). The number of days between semen processing and laboratory investigations did not negatively influence measured sperm concentration, semen volume, or TSC because the respective correlation coefficients were all small and nonsignificant.

Testosterone and fT declined with age in this relatively young population (*r* = −0.32, *p* = 0.001; and *r* = −0.38, *p* < 0.001, respectively); however, we found that neither SHBG, E_2_, LH, FSH, nor inhibin B or any sperm parameters were associated with age. Sperm characteristics and serum hormone and dioxin levels of participants are presented in Table 2. None of the participants had LH or SHBG levels outside the laboratory’s reference range (1–12 IU/L and 11–71 nmol/L, respectively). Three subjects had FSH values > 12 IU/L, 4 had an fT level below the laboratory’s lower reference value of 0.21 nmol/L, and 20 had a testosterone level < 11.1 nmol/L. The odds of having a testosterone value < 11.1 nmol/L significantly increased with the time of day blood was collected (OR = 1.3; 95% CI, 1.1–1.5; *p* = 0.005). Thirteen participants had a sperm concentration < 13.5 × 10^6^/mL and 23 had a sperm morphology value < 9% normal cells, criteria suggested by Guzick et al. (2001) to represent subfertility.

### Relationship between serum dioxin-like activity, food habits, and reproductive parameters

Dioxin-like activity increased significantly with age (*r* = 0.25, *p* = 0.02) and the monthly consumption of eggs (*r* = 0.25, *p* = 0.01), fish (*r* = 0.22, *p* = 0.03), and chicken (*r* = 0.16, *p* = 0.12, *n* = 97). In a linear model investigating the independent determinants of dioxin levels in blood, CALUX-TEQs rose by 2.9% for every year of age and by 5.2% and 3.7%, respectively, for every unit increase of egg and fish consumptions per month after exclusion of two cases—one reporting an egg consumption of more than once per day and one reporting eating fish daily (Table 3). Including these influential cases reduced the effect of a unit increase in egg and fish consumption to a change of serum dioxin levels of 1.1% (95% CI, −1.2 to 3.4%, *p* = 0.34) and 2.5% (95% CI, −0.4 to 5.6%, *p* = 0.09), respectively.

**Figure 1:**
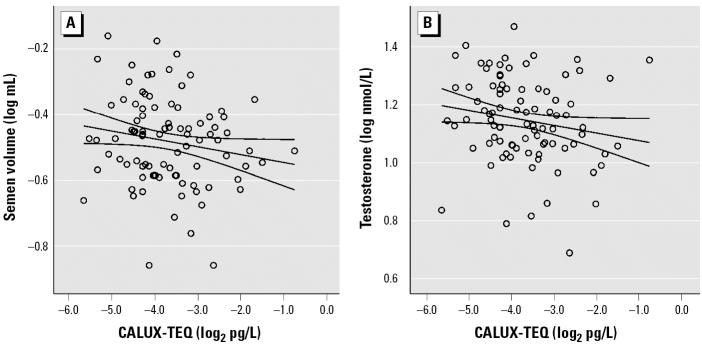
Association between CALUX-TEQs > 16 pg/L (*n* = 90) and (*A*) semen volume (*r* = −0.28, *p* < 0.01) or (*B*) testosterone (*r* = −0.24, *p* = 0.02).

In the whole population, serum dioxin-like activity was positively associated with sperm concentration (*r* = 0.23, *p* = 0.02) and negatively associated with semen volume (*r* = −0.23, *p* = 0.02) and total testosterone (*r* = −0.18, *p* = 0.07). No significant association was found for LH (*r* = −0.06, *p* = 0.53), FSH (*r* = 0.04, *p* = 0.66), testosterone/LH (*r* = −0.06, *p* = 0.54), or inhibin B (*r* = −0.06, *p* = 0.53), or for total sperm count (*r* = 0.06, *p* = 0.53) or sperm morphology (*r* = −0.02, *p* = 0.85). In a linear model, CALUX-TEQs > 16 pg/L (reducing the number of cases to 90) were associated with an increase in sperm concentration and a decrease in semen volume, total testosterone, and fT (Table 3, Figure 1). Exclusion of cases reporting a previous serious illness or surgery did not influence the regression coefficient of semen volume, but it increased the effect of dioxins on sperm concentration (percent increase for a 2-fold increase in CALUX-TEQs > 16 pg/L serum, 30.8%; 95% CI, 1.8–68.1%; *p* = 0.04). In the same reduced group, fT (but not total testosterone) was a significant determinant of semen volume but not of sperm concentration. In a linear model including age, days of abstinence, reported completeness of the semen sample, and sampling date, an increase of 10 pmol/L fT was associated with a 4.2% increase in semen volume (95% CI, 0.4–18.6%; *p* = 0.01; *n* = 90). Including CALUX-TEQs as an additional explanatory variable in the latter model reduced the percentage increase of semen volume by 10 pmol/L fT to 2.9%, and altered the percentage decrease of semen volume by a 2-fold increase of CALUX-TEQs to 12.4% (Table 3), rendering them both nonsignificant (*p* = 0.06 and 0.1, respectively).

## Discussion

We assessed serum levels of dioxin-like compounds in young men from the general population using the DR-CALUX assay, which incorporates aspects of exposure and biological effect. This bioassay-based approach yielded information reflective of life-long exposure, as well as more recent exposure through food consumption. More importantly, the biomarker was related to independently assessed but physiologically linked parameters of reproductive function in men.

Because of the resistance of TCDD and many other chlorinated hydrocarbons to degradation during global dispersion, they have become ubiquitous environmental contaminants, bioaccumulating in the food chain. In humans and wildlife, these compounds and their metabolites circulate in the blood and are stored in adipose tissue, and they have a half-life in the body of several years (ATSDR 2000; Bergman et al. 1994). Correspondingly, in the present study, CALUX-TEQ levels rose significantly with age. The Belgian population has historically been exposed to higher levels of dioxins and PCBs than many other Western populations (Liem et al. 2000; van Larebeke et al. 2001). Major improvements in industrial and waste incinerator processes have drastically decreased the emissions from point sources since the 1990s (Wevers et al. 2004; Wevers and Van Hooste 2004). Serum PCBs in adolescents were comparable or lower than the ones in other industrialized countries, whereas TEQ levels in older women were comparable with levels in Western countries 10 years earlier (Koppen et al. 2001; Nawrot et al. 2002). CALUX-TEQ levels in young adult males were lower than those reported in older women and, surprisingly, in adolescents (Koppen et al. 2002; Staessen et al. 2001). We did not measure individual PCB, PCDD, or PCDF congeners in the present cohort. However, although in pooled samples from the older women, highly significant correlations were found between the CALUX-TEQs and the chemically determined WHO-TEF–based TEQs (van den Berg et al. 2000), Koppen et al. (2001) concluded that further inter-laboratory validation is needed to improve the assay’s accuracy, making it a reliable tool allowing between-study comparison of results. Therefore, the present bioassay-based TEQs should be considered as relative measures of exposure within the present population.

We confirmed the effect of fish intake on the exposure of populations to dioxins and related compounds (Judd et al. 2004). However, the surprising significant role of egg consumption suggests a possible relationship with the January 1999 Belgian PCB and dioxin incident in which fats destined for animal feed production were contaminated with 40–50 kg of PCB-containing mineral oil (Bernard et al. 1999; van Larebeke et al. 2001). Subsequent PCB and dioxin measurements in Belgian foodstuffs showed that, both in absolute TEQ levels and the percentage of food samples, poultry and eggs were the most frequently contaminated; this was in line with the pathologic conditions found on chicken farms and recorded at the onset of the crisis (van Larebeke et al. 2001). The relationship between CALUX-TEQ levels and chicken consumption was positive, although not significantly.

In the present study, a 2-fold increase in serum dioxin-like activity was associated with a 25% increase in sperm concentration and a 16% decrease in semen volume, two interrelated parameters that were independently assessed (Table 3, Figure 1A). CALUX-TEQs, however, were not associated with the total sperm count or sperm morphology. These results suggest an interaction (direct or indirect) of dioxin-like substances with the secretory function of the seminal vesicles or the prostate (secretions from these glands constitute the main fraction of the ejaculate) but not with spermatogenesis. The functional integrity of the adult male accessory sex glands depends on androgens (Aümuller and Riva 1992; Aümuller and Seitz 1990). In addition, a number of their secretory proteins are under the direct transcriptional control of the androgen receptor, which is expressed in the secretory epithelium of these glands (Aümuller and Seitz 1990; Simon et al. 1997; Zhuang et al. 1992). In agreement, we found the biologically active fT to be a significant determinant of semen volume. Further, CALUX-TEQs correlated negatively with fT and testosterone (Table 3, Figure 1B). Although this might indicate an effect of dioxins on Leydig cell function, dioxin-like activity was not related to the testosterone/LH ratio, which is considered a marker of Leydig cell sensitivity (Andersson et al. 2004). In addition, no association was found with inhibin B, a marker of the functional state of the seminiferous epithelium (Andersson et al. 1998), further questioning the effects of dioxins on the testicular compartment. The fact that dioxin-like activity was not related to LH does not definitely exclude the possibility of an interaction at the pituitary level that is generally more difficult to assess because of the high intraindividual variation of LH secretion, which is under feedback control of estrogens and androgens.

Dioxins and/or PCBs have been shown to display estrogenic, antiestrogenic, and anti-androgenic effects (Gray et al. 2001; Safe 1995). However, our results are in line with the limited available *in vivo* experimental data on the reproductive effects of dioxin exposure during adulthood. Exposure of adult rats to TCDD at relatively high doses causes a decrease in ventral prostate and seminal vesicle weight that parallells and significantly correlates with a lower serum androgen status without affecting spermatogenesis (Johnson et al. 1992; Moore et al. 1985). In rodent Leydig cells, TCDD has been shown to decrease the secretion of testosterone through interference with LH receptor expression, impairment of the cholesterol mobilization to mitochondrial cytochrome P450scc, or reduction of the activity of this enzyme, thus interfering with the first steps in testosterone production (Fukuzawa et al. 2004; Moore et al. 1991; Moore and Peterson 1988). The lowered androgen levels in serum did not stimulate LH secretion because of an increased feedback responsiveness of the pituitary (Bookstaff et al. 1990).

Human data on adult exposure to dioxins or nondioxin AhR ligands and male reproductive and/or endocrine status are scarce. As part of the National Institute for Occupational Safety and Health (NIOSH) cohort, employees of two factories exposed to TCDD during trichlorophenol production for an average period of 2.7 years had significantly lower testosterone and higher LH and FSH with increasing dioxin levels up to 3,400 pg/g lipid (Egeland et al. 1994). Because low testosterone was not parallelled by high LH in the same individuals, the authors interpreted their data as not being suggestive of a role of dioxins in primary gonadal failure, but rather in a more subtle, possibly hormone receptor-related, physiologic mechanism. The approximately 10% decrease in testosterone over the whole dioxin concentration range (Egeland et al. 1994) seems smaller than the decrease we found in the present study, but the cohort was an average of 23 years older. Another exposed group consists of army veterans of Operation Ranch Hand who were involved in spraying the TCDD-contaminated defoliant Agent Orange during the Vietnam War. In the 1992 examination round, using a linear model similar to the one used in the present study, original and back-calculated 1987 serum TCDD levels were significantly negatively related to 1992 testosterone values but not to 1992 LH or FSH levels (Grubbs et al. 1995; Henriksen and Michalek 1996). These 1987 dioxin levels (median, 12.5 pg/g fat) (Grubbs et al. 1995) were lower than in the NIOSH cohort [median, 76 pg/g fat (Egeland et al. 1994)]. In both studies, levels < 20 pg/g fat (Operation Ranch Hand) or < 10 pg/g fat (NIOSH) were considered to represent background exposure. In a limited Australian study of 2,4,5-trichloro-phenoxyacetic acid sprayers, dioxin levels < 20 pg/g fat were inversely related to testosterone, but without accounting for the confounding role of BMI and age (Johnson et al. 2001). These three studies support the conclusion that testosterone levels decline with increasing TCDD over a wide concentration range.

The toxicologic profile of the complex PCB congener mixtures present in the environment is difficult to assess because of the multitude of the affected physiologic processes in the body and their sometimes interactive nature (ATSDR 2000), possibly leading to contradicting results in different epidemiologic studies. Hauser et al. (2003) grouped 57 PCB congeners according to their suspected biological activity in a cross-sectional study of 212 male partners in subfertile couples and found no association between dioxin-like or enzyme-inducing PCBs and sperm parameters, although there seemed to be a dose-related decrease in sperm motility and morphology for PCB-138. In two Swedish studies evaluating the endocrine and reproductive status in young men from the general population and in fisherman 24–65 years of age, PCB-153 was negatively associated with testosterone/SHBG ratio (a measure of fT) and/or with sperm motility (Richthoff et al. 2003; Rignell-Hydbom et al. 2004). Bush et al. (1986) found an inverse association between a calculated sperm motility index and the dioxin-like PCB-118 and the nondioxin-like PCB-138 and PCB-153 in seminal plasma, which reached significance only in those subjects with a sperm concentration below the WHO reference value of 20 million/mL (WHO 1992). In contrast, other less-comprehensive studies found positive relationships between sperm quality measures and individual non-AhR PCBs in men with idiopathic oligospermia and/or in controls (Dallinga et al. 2002; Magnusdottir et al. 2005). Unfortunately, we did not measure sperm motility or nondioxin-like PCBs in the present study. Further, more detailed studies are needed to investigate the relative or interactive role of dioxin-like and other PCBs in male fertility.

The mean percentage of normal sperm morphology in the present study was 14.7%, which is slightly higher than reported by Guzick et al. (2001) in fertile men using the WHO (1999) sperm morphology criteria. The median sperm concentration of 47 × 10^6^/mL in our population was similar to that in a cohort of young male military conscripts from Denmark (44 × 10^6^/mL), but lower than that reported in similar cohorts in Finland (61 × 10^6^/mL), Estonia (62 × 10^6^/mL), and Norway (53 × 10^6^/mL) (Jørgensen et al. 2002). Further, sperm concentration in the present study was significantly lower compared with men with proven fertility from Turku, Finland (81 × 10^6^/mL); Copenhagen, Denmark (60 × 10^6^/mL); Edinburgh, United Kingdom (76 × 10^6^/mL); and Paris, France (78 × 10^6^/mL) (Slama et al. 2002). In a German study on a self-selected sample of the general population (volunteers in clinical trials), Lemcke et al. (1997) reported a mean sperm concentration of 63 × 10^6^/mL. The comparison of sperm values across laboratories that do not share a standardized examination protocol is difficult (Jørgensen et al. 2002; Keel 2004). In addition, the comparability of the present population to the cohorts described above and the population’s representativeness for the general Flemish population is limited. It is a self-selected cohort (30% response rate) that was further reduced in number as a result of predefined exposure-related eligibility criteria (Muller et al. 2004). Nevertheless, the sperm concentration values we found are surprisingly low; further research should elucidate whether similar values are found in a nonselected sample from the general population, and thus if men in Flanders are among those with the lowest sperm quality in Europe.

In the present study, hormone values were as expected based on the laboratory’s reference range, except for the relatively high proportion of men with a low testosterone level. We showed this low testosterone level to be at least partly due to to the time of sampling, because of the diurnal secretory pattern of testosterone. Hormone–hormone and hormone–sperm relationships were in accordance with literature data obtained in normal healthy populations (data not shown).

Besides PCDDs, PCDFs, and some PCBs, a number of structurally divergent compounds bind to the dioxin receptor with varying affinity (Safe 1995). The chemically determined WHO-TEF–based TEQ could only explain 32% of the DR-CALUX variability in pooled samples from the older female cohort of the FLEHS (Koppen et al. 2001). In the present study, egg and fish consumption only explained 10% of the CALUX-TEQ variability. Therefore, a major drawback of this receptor-based analysis is that it leaves the questions of identity, relative importance, and source of the compounds underlying the response largely unanswered.

## Conclusion

To the best of our knowledge, this report is the first to link exposure to compounds with dioxin-like biological activity with reproductive parameters in nonoccupationally exposed men. Our data suggest that these compounds decrease testosterone levels in blood and interfere with the androgen-dependent secretions of the male accessory glands. Our observations do not indicate a role of dioxins in spermatogenesis. Nevertheless, secretory proteins from the seminal vesicles have been postulated to interfere with sperm capacitation and motility (Aümuller and Seitz 1990; Elzanaty et al. 2002). Sperm from men exposed to PCBs and/or PCDFs in adulthood displayed a significantly reduced capability of oocyte penetration (Hsu et al. 2003). Further research should therefore investigate whether the dioxin-related decrease of accessory gland secretion affects the fertilizing capacity of the spermatozoa.

## Figures and Tables

**Table 1:** Characteristics of the study population.

Characteristic	Participants (*n* = 100)
Age (years)	32.6 ± 5.8
Anthropometrics	
Body weight (kg)	80.1 ± 12.3
Body height (cm)	178.0 ± 6.7
BMI (kg/m^2^)	25.3 ± 3.6
Self-reported information	
Education [no. (%)]^a^	
Workers	35 (38)
Middle class	47 (50)
Educated professionals	11 (12)
Alcohol consumers [no. (%)]	56 (56)
Median alcohol consumption [g/day (IQR)]	8.2 (0.0–20.0)
Median passive smoking exposure [hr/day (IQR)]^b^	1.0 (0.0–3.0)
Men with earlier serious illness or operation [no. (%)]	11 (11)
Men taking medication [no. (%)]	30 (30)
Men with complete semen sample [no. (%)]	89 (89)
Median abstinence period [days (range)]	3.0 (0.0–15.0)
Food consumption	
Median meat servings per month (IQR)	20.0 (8.0–30.0)
Median fish servings per month (IQR)	3.0 (3.0–8.0)
Median milk servings per month (IQR)	30.0 (8.0–30.0)
Median egg servings per month (IQR)	8.0 (4.3–8.0)
Median chicken servings per month (IQR)	8.0 (3.0–8.0)
Median dietary animal fat intake [g/day (IQR)]	44.6 (32.4–55.1)
Blood fat (g/L)	6.1 ± 1.2

Data are mean ± SD except where indicated.

a *n* = 93 because of missing data.

b *n* = 84 because of missing data.

**Table 2:** Men’s sperm characteristics and hormone and dioxin levels.

	Median (IQR)	Mean ± SD
Sperm characteristics		
Sperm concentration (× 10^6^/mL)	47.1 (24.6–83.0)	61.5 ± 55.4
Semen volume (mL)	2.9 (1.7–4.1)	3.2 ± 1.8
TSC (× 10^6^)	126.6 (73.6–197.8)	155.3 ± 126.1
Sperm morphology (%)	14.0 (10.0–20.0)	14.7 ± 7.4
Hormone values and ratios		
Inhibin B (ng/L)	231.7 (182.6–263.6)	228.7 ± 60.1
FSH (IU/L)	4.2 (3.2–6.1)	5.1 ± 2.9
LH (IU/L)	4.4 (3.2–5.4)	4.5 ± 1.7
Testosterone (nmol/L)	14.0 (11.5–18.0)	14.8 ± 4.7
fT (pmol/L)	330.6 (311.2–351.1)	345.9 ± 106.9
E_2_ (pmol/L)	65.7 (55.2–78.7)	71.3 ± 32.2
SHBG (nmol/L)	23.7 (18.1–31.0)	25.4 ± 10.3
Testosterone/LH ratio (nmol/IU)	3.5 (2.6–4.6)	3.8 ± 2.0
Dioxin-like compounds in serum		
CALUX-TEQs (pg/L serum)	62.3 (43.3–110.1)	90.0 ± 85.4
CALUX-TEQs (pg/g fat)	11.9 (7.1–17.6)	14.6 ± 13.2

**Table 3:** Determinants of serum levels of dioxin-like compounds (CALUX-TEQs) and dose–effect relationships between CALUX-TEQs and reproductive parameters in men.

	Percent change (95% CI)	*p*-Value
Independent determinants of CALUX-TEQs^a^		
Years of age	2.9 (0.1 to 5.8)	0.04
Egg servings per month	5.2 (2.2 to 8.2)	0.001
Fish servings per month	3.7 (0.6 to 6.9)	0.02
Effects associated with increasing CALUX-TEQs^b^		
Sperm concentration^c^	25.2 (−1.7 to 59.5)	0.07
Semen volume^c^	−16.0 (−1.9 to −28.0)	0.03
Testosterone^d^	−7.1 (−0.5 to −13.2)	0.04
fT^d^	−6.8 (−0.4 to −12.7)	0.04

a Two men with outlying egg or fish consumption were excluded from the analysis. CALUX-TEQs are expressed as pg/L serum in a model additionally corrected for BMI and total grams of fat intake per day; effects are expressed as percentage change for a unit increase in the independent variable.

b Effect sizes were calculated for a 2-fold increase of CALUX-TEQ > 16 pg/L serum (*n* = 90).

c Effects additionally corrected for age, days of abstinence, reported completeness of the semen sample, and sampling date.

d Effects additionally corrected for BMI, age, and sampling date.
